# Schizophrenia and cortical blindness: protective effects and implications for language

**DOI:** 10.3389/fnhum.2014.00940

**Published:** 2014-11-28

**Authors:** Evelina Leivada, Cedric Boeckx

**Affiliations:** ^1^Department of Linguistics, Universitat de BarcelonaBarcelona, Spain; ^2^Catalan Institute for Advanced Studies and Research (ICREA)Barcelona, Spain

**Keywords:** schizophrenia, vision, protective effects, language, occipital cortex, thalamus

## Abstract

The repeatedly noted absence of case-reports of individuals with schizophrenia and congenital/early developed blindness has led several authors to argue that the latter can confer protective effects against the former. In this work, we present a number of relevant case-reports from different syndromes that show comorbidity of congenital and early blindness with schizophrenia. On the basis of these reports, we argue that a distinction between different types of blindness in terms of the origin of the visual deficit, cortical or peripheral, is crucial for understanding the observed patterns of comorbidity. We discuss the genetic underpinnings and the brain structures involved in schizophrenia and blindness, with insights from language processing, laying emphasis on the three structures that particularly stand out: the occipital cortex, the lateral geniculate nucleus (LGN), and the pulvinar. Last, we build on previous literature on the nature of the protective effects in order to offer novel insights into the nature of the protection mechanism from the perspective of the brain structures involved in each type of blindness.

## Introduction

The literature on psychiatric disorders of the past half a century contains a claim that relates congenital and/or early blindness (CB) to schizophrenia. The absence of reports of schizophrenic patients with CB has been repeatedly noted, and has led to the claim that CB confers a protective effect against schizophrenia (Chevigny and Braverman, 1950; Horrobin, 1979; Riscalla, 1980; Sanders et al., 2003; Silverstein et al., 2013a; Postmes et al., 2014). We will refer to this as the “Protection-Against-Schizophrenia” (PaSZ) hypothesis (Landgraf and Osterheider, 2013). This hypothesis predicts a decreased risk for developing schizophrenia in the blind population (for a schematic representation, see Landgraf and Osterheider, 2013: Figure 1).

The relevant literature includes various phrasings of this idea, ranging from milder claims about CB serving as a protective factor that reduces the risk to develop schizophrenia (Silverstein et al., 2013a) to stronger claims which imply that this risk is reduced to zero, as in Sanders et al. (2003) “no blind schizophrenics.” Regardless of the phrasing one adopts, most studies agree that “there has not been even one reported case of a congenitally blind person who developed schizophrenia” (Silverstein et al., 2013a, p. 1), and yet such cases should exist, on the basis of the joint probability rates calculated in Silverstein et al. (2013b), had it not been that a protection mechanism is in place^1^.

Although the protective effects of CB have been recently documented (see Landgraf and Osterheider, 2013; Silverstein et al., 2013a on possible cognitive changes as well as neurofunctional and multisensory reorganization effects), certain issues remain to be clarified. It is the aim of this paper to do so. For example, although there is a consensus concerning the fact that case-reports of patients with CB and schizophrenia have not been uncovered, there exist case-reports of congenital/early deafblindness, due to Usher syndrome, and psychosis (Dammeyer, 2011). Evidently, CB does not offer protection against neurodevelopmental disorders in general. For example, it has been suggested that blind children may present characteristics of infantile autism (see Carvill, 2001 for a review of different studies). People with Usher type I are congenitally deaf, start to lose vision early in life, and may develop schizophrenia (Dammeyer, 2012). The question is why adding deafness to the picture seems to lift the protective benefits and the reasons for this phenomenon are “not clear at present” (Silverstein et al., 2013a, p. 8).

In light of this, the present work aims to:

- present cases of co-existence of CB and schizophrenia, across different syndromes,
- draw attention to a point that is unaddressed in the relevant literature on blindness and schizophrenia and that becomes evident when one reviews the cases mentioned in (i): A distinction between different types of CB—cortical or peripheral (Dale and Salt, 2008)—established on the basis of the origin of the visual deficit, is crucial in order to explain why the protective effects appear to be lost in deafblindness. Peripheral CB (i.e., arising in the globe, CPB) seems to not confer any protective effects, as the case-reports presented in the next section suggest. Cortical CB (i.e., arising from lesions in the occipital cortex, CCB) presents a less clear picture: our research did not identify a case-report of CCB and schizophrenia, but absence of evidence is not evidence of absence. We suggest that the distinction between CCB and CPB is the key to explain the possible co-existence of schizophrenia and congenital (deaf)blindness in Usher and other syndromes. We further explore the genetic underpinnings of the observed patterns of comorbidity,
- relate schizophrenia and vision to language from the perspective of the brain structures recruited in each of these cognitive domains. Three structures stand out in this context: (a) the visual cortex, located in the posterior pole of the occipital cortex, which in CB individuals is also activated in language processing tasks (Bedny et al., 2011), (b) the lateral geniculate nucleus (LGN), part of the thalamus, which is the key structure related to the processing of information received from the retina, and (c) the thalamic pulvinar, a structure with connections to visual processing (Robinson and Petersen, 1992), but also to language (Llano, 2013) and shape abnormalities in schizophrenia (Thong et al., 2013).

The paper is organized as follows: In Section Cases of Congenital Blindness and Schizophrenia, the distinction between CCB and CPB is presented and the reasons for assuming it are discussed. The predictions of this distinction are then put in perspective by presenting various case-reports. In Section Genetic Underpinnings and Brain Structures, we explore the genetic parallels of the patterns of comorbidity observed at the phenotypic level. Section The Brain Basis of the Protective Effects addresses the origin of the protective effects in relation to the CCB/CPB distinction and the brain basis of each type of blindness. In Section Blindness, Schizophrenia, and Language, the focus is on the brain structures involved in blindness and schizophrenia also with insights from language processing. Section Conclusions concludes.

## Cases of congenital blindness and schizophrenia

The World Health Organization estimated that in 2002 there were 161 million persons worldwide with visual impairment, with 37 million of them being blind (i.e., visual acuity less than 3/60; see Resnikoff et al., 2004). An estimated 1.4 million were blind children below the age of 15 years (Resnikoff et al., 2004). Other research suggests that these numbers are higher: 259 million persons with visual impairment worldwide, including 42 million blind persons (Dandona and Dandona, 2006). According to the World Health Organization, schizophrenia affects about 24 million people worldwide^2^.

Despite these large numbers of prevalence for each one of the two conditions, it has been argued that patients with CB seem to be “immune” to schizophrenia, since numerous studies have failed to produce even one well-defined case (Sanders et al., 2003). The aim of this section is to present such cases. We suggest that two factors are necessary in order to delimit the origin of the protective effects: (a) age of blindness onset (congenital/early vs. late) and (b) origin of visual deficit (cortex vs. globe). The PaSZ hypothesis distinguishes between two factors as well: (a) age of blindness onset and (b) degree of visual capacity (i.e., blindness, impairment, normal vision, highly trained vision, etc.). The second factor is indeed relevant in the context of Landgraf and Osterheider (2013), where the relative risk for schizophrenia is related to a continuum of visual capacity toward a “peak risk” (see also Landgraf et al., 2012; Silverstein et al., 2013a). Both sets of factors enter into the relation between schizophrenia and blindness, but in the context of the present discussion the second factor of the PaSZ hypothesis is not so crucial, because we focus on the one edge of the continuum (i.e., blindness).

As the case-reports presented in this section show, only congenital/early *cortical* blindness—the type of blindness that occurs when bilateral lesions of the occipital cortex deprive the individual from vision (Cummings and Trimble, 2002, p. 110)—seems to confer protective effects. A factor to be taken into consideration is the lack of uniformity in defining age of onset of early blindness. In the context of our study, the distinction between congenital/early vs. late blindness is crucial; therefore, concurring with a long line of research, we take the age of onset of congenital blindness to be birth and the mean age of onset of early blindness to be <5–6 years of age (Arno et al., 2001; Kitada et al., 2013). Following the practice in previous studies (e.g., Silverstein et al., 2013a), these two groups are merged and collectively referred to as CB.

The case-reports and their analysis examined in the present work are the result of extensive text-mining and database searching through PubMed, OMIM, String 9.1 as well as individual journal search for all results retrieved by searches of any combination of the terms “schizophrenia,” “psychosis,” “congenital/early/childhood/adolescent/late/peripheral/ocular/cortical/cerebral blindness.” Our searches were not constrained by any time frame or language-/ethnic-group restrictions.

Table 1 shows case-reports for three out of four groups of blindness that arise when we combine the two factors: age of onset (CB vs. late blindness) and origin of the visual deficit (cortical vs. peripheral). Although we identified cases of CB and schizophrenia, they are all of the CPB type. Our research did not identify a single case of CCB and schizophrenia, although in one case presented in Table 2 (i.e., Stewart and Sardo, 1965) the origin of the deficit is not reported.

**Table 1:** **Case-reports across types of blindness**.

**Types of blindness**	**Late peripheral blindness (LPB)**	**Late cortical blindness (LCB)**	**Congenital/Early peripheral blindness (C/EPB)**	**Congenital/Early cortical blindness (C/ECB)**
Schizophrenia	✓	✓	✓	?
Case-reports	Checkley and Slade, 1979	Engel et al., 1978	Gobetz, 1967	
	Ogden, 1982	Sonavane et al., 2012	Weiss et al., 1981	

**Table 2:** **Features of case-reports**.

**Case-reports**	**Gender**	**Age of onset (years)**	**Origin/Type of visual deficit**	**Type of blindness**	**Psychotic features**	**Other remarks**
Checkley and Slade, 1979	F	42	Retinitis pigmentosa	LPB	Hallucinations	Partial hearing loss
Ogden, 1982	M	>16	Retinitis pigmentosa	LPB	Hallucinations, delusions	-
Grøndahl and Mjøen, 1986	M	62	Retinitis pigmentosa	LPB	Yes	Psychosis is reported, but not schizophrenia Usher type I (deafblindness)
Engel et al., 1978	F	20	Cortex	LCB	Catatonia	Partial complex status epilepticus^3^
Sonavane et al., 2012	F	18	Cortex	LCB	Yes	ECT-induced blindness
Sharfstein et al., 1999	F	>60	Cortex	LCB	Yes	Psychosis is reported, but not schizophrenia Severe dementia
Gobetz, 1967	F	Birth	Optic atrophy	CPB	Hallucinations	-
Weiss et al., 1981	F	≃6	Retinitis pigmentosa	EPB	Catatonia	Laurence-Moon-Bardet-Biedl syndrome
						Catatonic schizophrenic-like psychosis
Kerschbaumer, 1943	F^4^	Birth	Eye under-development	CPB	No information	3 cases are reported
Kay and Roth, 1961	F	<82	No information	late	Delusions	Partial hearing loss
Stewart and Sardo, 1965	F	Birth	No information	Congenital	Catatonia	-

In Table 2, the relevant case-reports are presented in further detail. Unless stated otherwise, all case-reports involve a diagnosis of schizophrenia.

Table 2 presents five cases of CPB and one case of EPB. Starting off from Stewart and Sardo (1965), this is the only case of schizophrenia and CB brought up in Silverstein et al. (2013a) and the validity of the schizophrenia diagnosis is questioned on the basis of absence of psychotic symptoms. However, it can be observed that the patient shows disorganized speech and grossly disorganized behavior. On this basis, diagnostic criterion A for schizophrenia of DSM-5 (American Psychiatric Association, 2013) is met even in the absence of delusions or hallucinations^5^. Second, the patient does seem to show psychotic symptoms that meet DSM-5 diagnostic criteria for catatonia, as these are reviewed in Tandon et al. (2013: Table 1). More specifically, (i) echolalia, (ii) agitation, and (iii) phases of mutism are present in the patient's behavior. The origin of the visual deficit is not explicitly reported, but crucially there is evidence that blindness run in the family. Siblings of the patient are reported to be “afflicted with the same congenital blindness” (Stewart and Sardo, 1965, p. 125). On this basis, we are inclined to assume this is a case of hereditary blindness that involves the globe (i.e., CPB).

The second case-report that is quite explicit on the existence of psychotic symptoms is given in Gobetz (1967). Even if Silverstein et al. (2013a) are right in questioning the schizophrenia diagnosis in Stewart and Sardo (1965), our research has uncovered case-studies where both the psychotic features and the peripheral origin of CB are explicit; the case-report presented in Gobetz (1967) is one example. The cause of CB is optic atrophy. The patient was first hospitalized in the age of 15 and the medical reports make reference to psychotic features such as hallucinations and paranoid thinking. The extensive reference to the contents of her hallucinations leaves no doubt that this is indeed a case of comorbidity between CPB and schizophrenia, with psychotic features present.

Third, Kerschbaumer (1943) presents three cases of schizophrenia and CB. The cause of blindness is eye underdevelopment. Kerschbaumer does not offer the profile of the three congenitally blind patients in terms of psychosis. However, the comments offered in the Conclusion Section (point 9) of that medical report do not offer any reason for challenging the diagnosis of schizophrenia. More specifically, Kerschbaumer draws attention to the need to avoid the misdiagnosis of the catatonic type of schizophrenia as manic-depressive psychosis or the misdiagnosis of early schizophrenia with predominant somatic delusions as psychoneurosis with paranoid ideation. It seems that the diagnoses of schizophrenia have been made with the necessary caution so as to not misrepresent cases falling within the schizophrenia spectrum disorders as schizophrenia. On this basis, we do not have any reason to question the diagnosis of schizophrenia that is given in Kerschbaumer (1943).

Last, Weiss et al. (1981) report a patient who at the age of 6 (upon going to school for the first time) was found to be “almost completely blind” (p. 259) and transferred to a special school. This case is less clear than the ones presented above for two reasons: first, the age of blindness onset is not birth, and second, the patient is almost blind, but not completely. Putting this case-report in the context of the PaSZ hypothesis, almost complete blindness should fall within the low-risk zone for developing schizophrenia identified in Landgraf and Osterheider (2013: Figure 1). With respect to the age factor, the absence of consensus across studies with respect to the age range that corresponds to “early blindness” makes it hard to unambiguously say whether 6 years of age classifies as early or late blindness. Depending on where one puts the limit on the age range, the patient in Weiss et al. (1981) may or may not count as early blind schizophrenic.

Overall, the conclusion to be drawn from these case-reports is that there are cases of comorbidity between congenital and early PB (i.e., CPB) and schizophrenia, but so far no such case has been discovered for congenital or early CB (i.e., CCB) and schizophrenia.

Usher syndrome is in part the ground on which Silverstein et al. (2013a) comment on cases of congenital deafblindness by arguing that it is not clear at present why adding deafness to a case of congenital blindness lifts the protective effects against schizophrenia. As shown in Table 2, not all cases of CB and schizophrenia involve hearing loss. In light of this, deafness cannot be viewed as lifting the protective effect, since the latter appears lifted in cases that deafness is not reported. According to the US National Institute on Deafness and Communication Disorders, Usher syndrome is the most common condition affecting both vision, via retinitis pigmentosa, and hearing^6^. In the literature, one finds numerous reports on the relation between retinitis pigmentosa and schizophrenia (Shukla et al., 1983; McDonald et al., 1998). Recently, an association between usherin and psychotic symptoms has been established on the basis of a genetic analysis of two siblings with Usher syndrome and schizophrenia (Domanico et al., 2012).

Bardet-Biedl syndrome presents a similar picture. It has been referred to as Laurence-Moon-Bardet-Biedl, but Laurence-Moon and Bardet-Biedl are now recognized as separate entities, although not always (Moore et al., 2005). Blindness that is the result of childhood-onset retinitis pigmentosa and schizophrenia-like psychosis can co-exist in Bardet-Biedl syndrome (Weiss et al., 1981). The main symptoms of this syndrome are rod-cone dystrophy (atypical retinitis pigmentosa), postaxial polydactyly, mental retardation, hypogonadism, and renal dysfunction (Green et al., 1989; Beales et al., 1999). Language delays have been observed too (Chen et al., 2004; Aloulou et al., 2011). Case-reports indicate comorbidity between (Laurence-Moon-)Bardet-Biedl and schizotypal traits: Delusions of persecution have been reported (O'Mahony, 1954), but also full-blown schizophrenia (Jain and Garg, 1981).

Disrupted-in-Schizophrenia1 (*DISC1*) is one of the most frequently discussed susceptibility loci for schizophrenia. It could be described as the standard textbook example of a candidate gene for vulnerability in schizophrenia (e.g., Black and Andreasen, 2011, p. 78). Numerous studies have found positive evidence of association between schizophrenia and genes encoding DISC1-interacting proteins across a variety of ethnic groups (Bradshaw and Porteous, 2012). Crucially, *DISC1* plays a “role in radial neuronal migration via anchoring dynein motor-related proteins to the centrosome, including NDEL1, BBS1 and BBS4, two of the proteins mutated in Bardet-Biedl syndrome” (Ishizuka et al., 2011, p. 92; see also Kamiya et al., 2005). In other words, the genetic basis of Bardet-Biedl suggests that this is a syndrome that may manifest comorbidity between schizophrenia and retinitis pigmentosa.

Our review of case-reports in different syndromes suggests that schizophrenia and, more generally, schizotypal features can be found in the CB population as long as the cause of blindness is found in the globe. The situation is less clear in cases of CCB; in the absence of case-reports that involve both CCB and schizophrenia, the PaSZ hypothesis (Landgraf and Osterheider, 2013) can be maintained. Aiming to understand the genetic underpinnings of the patterns of comorbidity presented in this section, the next section elaborates on the genetic underpinnings of these patterns and the brain structures involved in the interplay between blindness and schizophrenia.

## Genetic underpinnings and brain structures

Various case-reports listed above suggest a pattern of comorbidity between retinitis pigmentosa and schizophrenia. This suggests that a potentially interesting connection might be found at the genetic level. In order to investigate the latter, we identified risk genes with high prevalence for each condition, schizophrenia and CPB (due to either retinitis pigmentosa or anophthalmia). The relevant sets of genes were then fed to String 9.1, a database dedicated to known and predicted protein-protein interactions (Szklarczyk et al., 2011). The resulting networks that represent these interactions integrate information from a variety of sources, “thus acting as a meta-database that maps all interaction evidence onto a common set of genomes and proteins” (Jensen et al., 2009).

### Results

**Figure 1:**
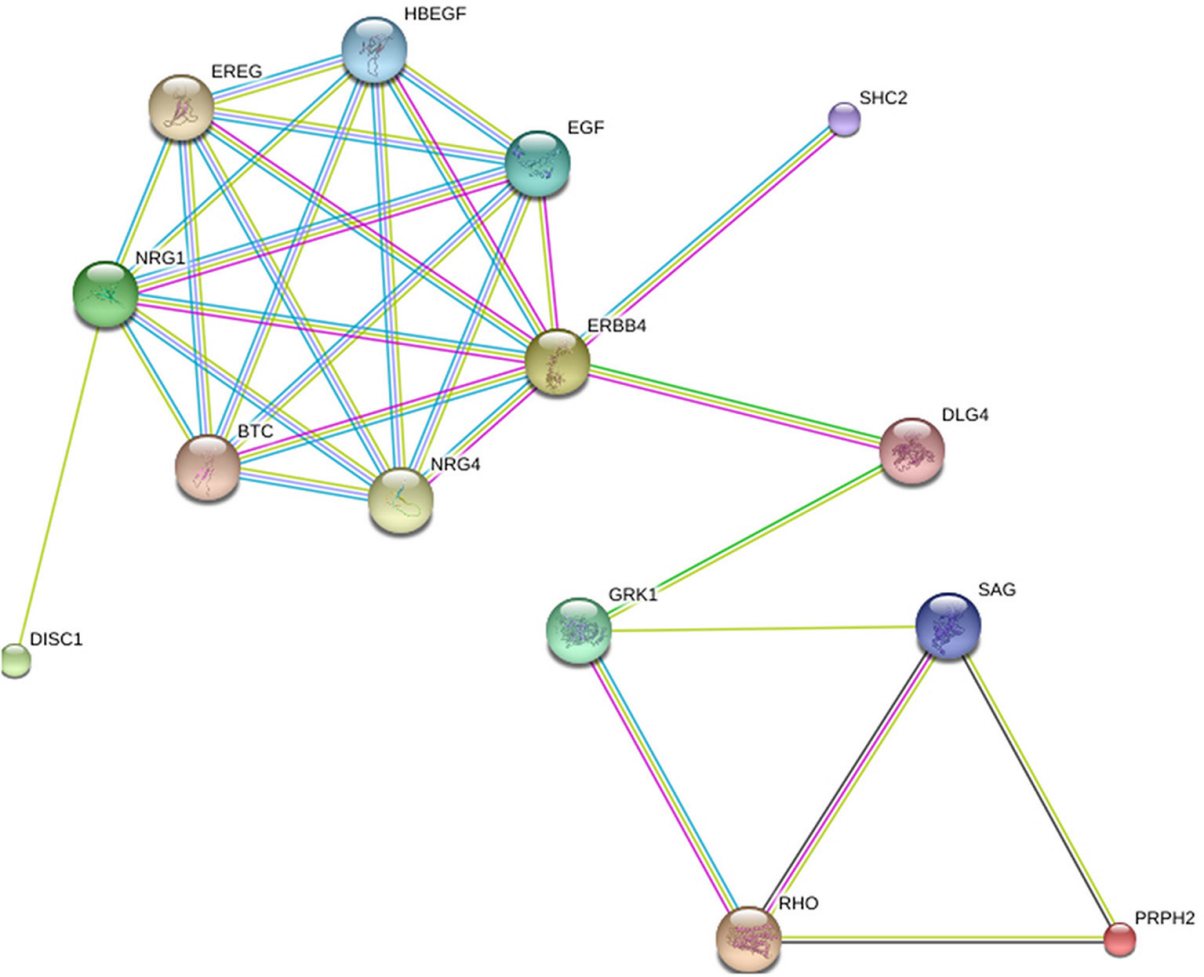
**The relation between RHO, PRPH2, DISC1 and ERBB4**. Generated by String 9.1.

In Figure 1, the relation depicted is between two genes that cause retinitis pigmentosa on the one hand and two genes that are implicated in schizophrenia on the other. The first group consists of *RHO* and peripherin 2 (*PRPH2*); two genes with high prevalence according to Sullivan et al. (2006). The second group involves *DISC1* and *ERBB4*; the latter being the gene with the highest ranking in terms of strength of association with schizophrenia GWAS data sets among the oligodendroglial and myelin related genes (Roussos and Haroutunian, 2014).

The networks are presented in the evidence view. The medium confidence value is 0.400. Different lines between nodes represent different types of evidence for the reported association (i.e., black is for co-expression, dark blue for co-occurrence, light blue for databases, dark green for neighborhood, light green for text mining, pink for experiments, red for gene fusion, purple for homology). Protein nodes are colored and sized randomly.

In Figure 1, two genes are found to establish the connection between *RHO* and *ERBB4*, each of them a gene with connections to schizophrenia and retinitis pigmentosa, respectively: *DLG4* and *GRK1*. *DLG4* encodes a member of the membrane-associated guanylate kinase family and has been recently argued to play a role in schizophrenia pathogenesis (Balan et al., 2013). Other studies did not detect any mutations at the protein-coding sequences of *DLG4* in schizophrenia populations, but found associations that suggest that the expression of *DLG4* is tied to the susceptibility of schizophrenia (Cheng et al., 2010). *GRK1* (G-protein-coupled receptor kinase 1) encodes a member of the guanine nucleotide-binding protein (G protein)-coupled receptor kinase subfamily of the Ser/Thr protein kinase family. It has been associated primarily with night blindness or, else, nyctalopia (Azam et al., 2009), which is a symptom of retinitis pigmentosa (Sharma et al., 2004).

**Figure 2:**
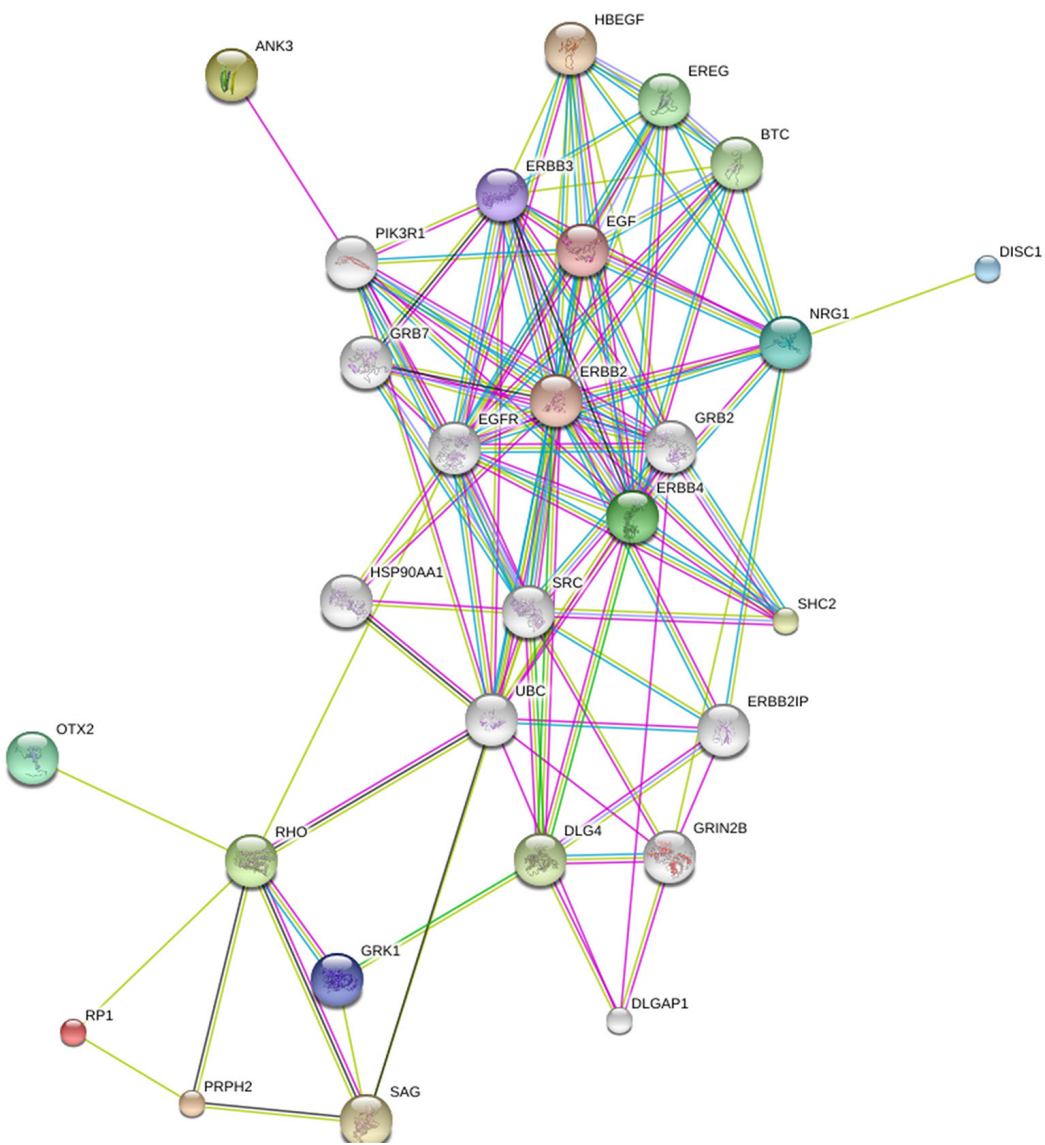
**The relation between RP1, RHO, PRPH2, DISC1, ERBB4, ANK3, NRG1, and OTX2**. Generated by String 9.1.

In Figure 2, *OTX2, RP1, ANK3*, and *NRG1* have been added to the group of four genes presented in Figure 1. We think *OTX2* stands out because it appears to play a role both in CPB and schizophrenia. It has been implicated in the development and function of the retina (Bovolenta et al., 1997; Baas et al., 2000; Martinez-Morales et al., 2001) and also in CPB due to anophthalmia (Bridge et al., 2009). Otx2 homeoprotein coordinates postnatal parvalbumin (PV) cell development and activates visual cortical plasticity (Sugiyama et al., 2008). Both structural and molecular alterations of PV neurons and protein levels have been found in schizophrenia subjects (Lewis et al., 2012; Glausier et al., 2014). *OTX2* is also related to thalamic development (Scholpp and Lumsden, 2010) and “dysfunctional thalamus-related networks” have been reported in schizophrenia (Pinault, 2011). *RP1* completes the triad (*RHO, PRPH2, RP1*) of genes with highest prevalence for retinitis pigmentosa (Sullivan et al., 2006). *ANK3* and *NRG1* have the second and third highest ranking respectively in terms of strength of association with schizophrenia GWAS data sets among the oligodendroglial and myelin related genes (Roussos and Haroutunian, 2014).

The conclusion to be drawn is that the patterns of comorbidity that are observed between CPB and schizophrenia at the phenotypic level are related to links between susceptibility loci for the two disorders at the genetic level. In other words, genes that may be implicated in CPB show multiple connections to genes that are often referred to as schizophrenia candidate genes.

So far, an attempt was made to match the connections observed at the genetic level to patterns of comorbidity between CPB and schizophrenia observed at the phenotypic level. Given that the highly heterogeneous nature of schizophrenia could be caused by multiple rare alleles (McClellan et al., 2007), the genetic underpinnings of the disease are definitely worth exploring, but provide only a part of the overall picture. Indeed, many recent reviews argue that the dysconnectivity between brain regions is the place to look for the causes that underlie cognitive abnormalities in schizophrenia (Fitzsimmons et al., 2013).

For example, the dysconnectivity between brain regions in schizophrenia has been related to—among others—white matter abnormalities/hyperintensities (Fitzsimmons et al., 2013; Hahn et al., 2014), gray matter volume reduction (Hulshoff Pol et al., 2002), abnormalities in the anterior cingulate cortex activity (Sanders et al., 2002), “volumetric alterations in prefrontal-thalamic-cerebellar gray matter networks” (Rüsch et al., 2007), “aberrant inter-hemispheric connectivity of anterior cortical and sub-cortical brain regions” (Hulshoff Pol et al., 2004), “dysfunctional thalamus-related networks” (Pinault, 2011), and “impaired visual cortical plasticity” (Çavuş et al., 2012). It is interesting to note here that the observed dysconnectivity between brain regions is not restricted to schizophrenia, but rather is a characteristic of psychosis and, as such, it is shared across the (schizophrenia spectrum) disorders that involve psychosis. Individuals with non-clinical psychosis (i.e., healthy individuals that experience occasional psychotic symptoms but are not schizophrenics) show reduced connectivity compared to controls between regions in all of the networks and the thalamus (Orr et al., 2014). Positive symptoms were associated with “increased connectivity between the cingulo-opercular network and visual cortex” (Orr et al., 2014). In sum, there is growing support for the existence of aberrations in connectivity between different networks and brain structures in individuals with psychosis. Two of the structures recurrently showing up in the relevant literature are the visual cortex and the thalamus, a point to which we now turn.

## The brain basis of the protective effects

As the presentation of different syndromes in Section Cases of Congenital Blindness and Schizophrenia suggested, a link seems to exist between visual dysfunction of *peripheral* origin and schizophrenia. This relation is evidenced also in first-degree relatives of patients with schizophrenia (e.g., in forms of eye movement dysfunction; Calkins et al., 2008). The aim of this section is to explore the different brain bases of the two types of blindness, CCB and CPB, and offer an explanation as to why the protected effects seem conferred to a greater degree by the former, since we do find cases of comorbidity of the latter with schizophrenia.

One explanation as to why the protective effects appear to be granted differently in CCB and CPB could be related to the fact that the two types of blindness have a different brain basis. In other words, the protective effects could be the outcome of impaired connectivity between brain areas disrupted differentially in CCB and CPB. The basis of this discussion lies in the idea that the protective effects are not the result of blindness *per se*, but rather of brain changes that occur secondary to blindness (Silverstein et al., 2013a, p. 1). Naturally, since the two types of blindness, cortical and peripheral, have a different brain basis, the subsequent brain changes may not be uniform, and a modulation of the protection mechanism may be allowed across the two.

Importantly, the modulation of the protection mechanism that we assume to exist is compatible with the idea in Silverstein et al. (2013a) that a cluster of perceptual and cognitive functions, which are impaired in schizophrenia and significantly enhanced in congenital blindness, might offer protective effects (see also Cattaneo et al., 2008; Cattaneo and Vecchi, 2011 for reviews of enhanced perceptual skills in the CPB population). Because the enhancement comes from brain changes secondary to experiencing the world without vision, it is highly likely that the protective effects are to be found in both CPB and CCB, because in both conditions the world is experienced without vision. The modulation we propose boils down to the protection mechanism being further reinforced in cases of CCB due to the brain basis of this condition.

The idea of perceptual organization dysfunction often manifested in schizophrenia through object fragmentation is relevant in this respect (Uhlhaas and Silverstein, 2005; Silverstein and Keane, 2011a). This idea supports a view of schizophrenia as “fundamentally a cognitive (i.e., information processing) disorder” (Silverstein et al., 2013a, p. 1). The brain structure that appears to be most critical for information processing is the thalamus; abnormal thalamic structures and dysfunctional thalamus-related networks have been repeatedly implicated in schizophrenia (Andreasen, 1997; Pinault, 2011). The CCB/CPB distinction is highly relevant in this context. In cases of CPB due to anophthalmia, significant white matter volume reductions have been observed around the thalamus (Bridge et al., 2009). The LGN in the anophthalmic subjects was found to be abnormal, compared to the controls; however, the connectivity between the LGN and the primary visual cortex did not differ between the two populations (Bridge et al., 2009). The conclusion drawn was that “these findings could imply a rewiring of brain function in the occipital lobe at a subcortical in addition to or instead of at a cortical level” (p. 3479).

In another study, the optic pathways of patients with CPB showed atrophy or hypoplasia in 7 of the 12 patients (58%), the optic nerve in 4 patients, the chiasm in 4 patients and the tract in 2 patients, however in all 12 patients the visual cortex appeared morphologically normal (Breitenseher et al., 1998). The picture is different in cases of CCB, where subcortical structures are intact and have been reported to be activated for eye contact (Burra et al., 2013). This subcortical neural pathway, which leads from the retina to the superior colliculus, to the pulvinar and then to the amygdala, permits processing of potentially threatening stimuli (Skuse, 2006). Observing first that schizophrenia has been described as a processing disorder that is related to dysfunctional thalamic networks and second that the protective effects appear to be limited to the type of blindness that does not imply thalamic abnormalities, it is possible that the protective effects could boil down to functions of the—rewired in the case of CB—subcortical (thalamic) structures that are largely intact in CCB, but not in CPB.

Usually the language faculty is not part of discussions on the relation between blindness and schizophrenia. However observing the interesting connections between blindness and language noted in Silverstein et al. (2013a), the next section builds on the insights offered in that work in order to discuss the “blindness-schizophrenia-language” triangle from the perspective of the brain structures involved in vision, schizophrenia, and language processing.

## Blindness, schizophrenia, and language

Yoon et al. (2013) start their review of studies of visual perception in schizophrenia by drawing our attention to the “wide variety of processes that have implicated abnormalities at various levels of the visual system, from the retina (Balogh et al., 2008) to higher-order extrastriate visual cortex (Butler et al., 2008).” The intricate relation between (absent, present, and impaired) vision and schizophrenia has been thoroughly reviewed recently (e.g., Landgraf and Osterheider, 2013; Silverstein et al., 2013a; Yoon et al., 2013) and the present section does not need to address this relation further. Instead, the aim is to add another factor to the equation: language.

The relation between schizophrenia and language has at times been approached in terms of the former being a disruption of the latter. Thought disorder has been described as “the most studied symptom of a much-studied disorder, schizophrenia” (McKenna and Oh, 2005, p. ix). There is a long tradition that views thought disorder as a disorder of language (Kleist, 1914) or, in more nuanced terms, as language abnormality making a contribution to the phenomenon of thought disorder in schizophrenia (McKenna and Oh, 2005, p. 99). Although there is no doubt that some aspects of language may be disrupted in schizophrenia, it is crucial to realize that these linguistic abnormalities might be due to a cognitive abnormality manifested in a linguistic dress (for example, the increased use of second person pronouns in schizophrenia that (Watson et al., 2012) report could be due to perturbations in non-egocentric referencing which are demonstrated in non-linguistic tasks, e.g., Landgraf et al., 2010).

Silverstein et al. (2013a) provide a brief overview of language delays in CB schizophrenics and they make a crucial observation. Noticing abnormalities related to language processing in CB children (Perez-Pereira and Conti-Ramsden, 1999), they propose that “[o]ne possible consequence of these is a reduction in the risk of developing disordered thought, which is commonly seen in schizophrenia. For example, many thought-disordered patients with schizophrenia have a tendency toward overabstraction and overinclusion in their thinking, as well as overly elaborated (and more easily primed) semantic networks (Siekmeier and Hoffman, 2002; Lerner et al., 2012)” (p. 3). CB children have the opposite tendency in being “characterized by a lack of over-generalization of concepts and categories, and a reduced number of word inventions (Andersen et al., 1984, 1993)” (p. 4).

One conclusion to be drawn from this state of affairs is that CB controls or suppresses the overabstraction tendencies and the abnormal associative processes (i.e., fragmentation) that Lerner et al. (2012) report in relation to patients with schizophrenia. This control can be thought of as part of the protective effects that CB confers. Such an observation makes the “blindness-schizophrenia-language” triangle worth analyzing further, mainly from the perspective of brain structures identified in the previous section, which is something that has not been done so far in the literature on blindness and schizophrenia.

Back to vision, Amedi et al. (2005) describe the process of seeing along the following lines: “Focused light landing on the retina causes neuronal signals to leave the eye through the optic nerve; those signals are sent via the LGN of the thalamus to the occipital cortex, where the majority of visual processing takes place” (p. 306). There is experimental support for the claim that some brain areas switch functions; one of them is the function of the occipital cortex in the blind population. More specifically, the visual cortex of peripherally blind subjects is recruited for the processing of tactile and auditory information (Sadato et al., 1996, 1998; Pascual-Leone and Hamilton, 2001). Also, occipital cortex activation has been reported during speech/language processing and semantic-judgment tasks (Roder et al., 2002; Burton, 2003; Pascual-Leone et al., 2005; Bedny et al., 2011). It has been suggested that recruitment of visual circuits for language processing depends on the age factor: the presence of blindness during childhood (Bedny et al., 2012).

With respect to language processing skills, patients with CPB are significantly better than sighted individuals in ultra-fast speech perception. In a recent experiment, among the areas that showed high activation were the right-hemispheric primary visual cortex, the contralateral fusiform gyrus, and the bilateral pulvinar (Dietrich et al., 2013). The fusiform gyrus is argued to subserve face perception (Kanwisher et al., 1997), an ability impaired in schizophrenia (Chen et al., 2008). Viewing blindness, schizophrenia, and language altogether, from the perspective of the brain structures that seem to play a more leading role compared to others, three structures figure prominently: (a) the visual cortex, located in the posterior pole of the occipital cortex, (b) the LGN, and (c) the pulvinar. These three structures are intimately interconnected in visual processing: The primary visual cortex receives input via LGN, while the lateral pulvinar nucleus projects to the visual cortex as well and “is able to powerfully control and gate information outflow” from the primary visual cortex (Purushothaman et al., 2012, p. 905).

With respect to schizophrenia, all three structures stand out as key points for abnormalities. There is a variety of studies reporting impaired plasticity, dysfunctional network connectivity, and a disturbed shape due to disrupted corticogenesis in relation to the visual cortex of schizophrenic individuals (Çavuş et al., 2012; Schultz et al., 2013; Ford et al., 2014). With respect to the two thalamic structures discussed here, LGN is one of the structures where Protocadherin-11 (PCDH11) is expressed in the neonatal brain (Matsunaga et al., 2013, Table 1). Crucially, this gene has been related to psychosis (Crow, 2013). The volume of LGN was found to be increased in patients suffering from mood disorders such as major depression or bipolar disorder with psychotic features, although this increase was not found in the schizophrenic group (Dorph-Petersen et al., 2008). True, other studies relate LGN to abnormalities distinct to schizoaffective disorder (Smith et al., 2011). However, when putting these findings in the bigger picture, one should take into account that the dividing lines among schizophrenia spectrum disorders are not always clear. Overall, it seems that the population may vary across studies, however it consistently targets a disorder that involves psychosis. Lastly, thalamic shape abnormalities in schizophrenics have been found also in regions related to pulvinar and medial dorsal nuclei (Thong et al., 2013).

In the case of language, the role of the visual cortex in language tasks has been demonstrated across studies, as discussed earlier in this section. Reports on the relation between language and the thalamus date back more than a century (Dejerine and Roussy, 1906). Recently, the thalamus was argued to be central to human cognition, and particularly to language, on the basis of a new candidate gene set whose members are related both to the development of the thalamus and to language-associated cognitive disorders (Boeckx and Benítez-Burraco, 2014). With respect to LGN, PCDH11 is argued to play a critical role in aspects of human cognition related to language (Crow, 2002; Priddle and Crow, 2009, 2013). Moreover, a number of reviews points to a possible involvement of the thalamus in manipulations of lexical information and naming processes, laying emphasis on the role of the pulvinar (Hebb and Ojemann, 2013; Llano, 2013).

## Conclusions

It has been argued that vision science can significantly advance our understanding of schizophrenia (Silverstein and Keane, 2011b). The aim of this work was to explore aspects of the relation between absent vision and schizophrenia. The departure point was the claim that CB confers a protective effect against schizophrenia; a claim based on the absence of case-reports of comorbid CB and schizophrenia (Chevigny and Braverman, 1950; Horrobin, 1979; Riscalla, 1980; Sanders et al., 2003; Landgraf and Osterheider, 2013; Silverstein et al., 2013a; Postmes et al., 2014). We presented a number of relevant case-reports and showed that all fall into a specific type of blindness, that of peripheral origin. On this basis, we suggested that the distinction between different types of blindness in terms of the origin of the visual deficit is crucial for understanding both the observed patterns of comorbidity and the nature of the protective effects. Building on previous work on the topic (Silverstein et al., 2013a), we argued in favor of a modulation of the protection mechanism in cases of congenital/early cortical blindness.

In our research we did not manage to uncover any co-occurrence of congenital/early cortical blindness and schizophrenia. Although we acknowledge, together with Silverstein et al. (2013b) and Landgraf and Osterheider (2013) that absence of evidence is not evidence of absence, we are inclined to assume on the basis of the prevalence rates of each condition, that some case-reports should have been identified, had it not been for some protective effects being in place. It is likely that future research on the topic will shed more light on the nature of the protective mechanism, possibly clarifying whether the protective effect is confined to schizophrenia or extends to schizotypal traits more broadly.

### Conflict of interest statement

The authors declare that the research was conducted in the absence of any commercial or financial relationships that could be construed as a potential conflict of interest.

## Footnotes

^1^Silverstein et al. (2013b) calculated that the joint probability of a person having both CB and schizophrenia, if the two are independent, would be 0.02% or 2 out of every 10,000. They do now draw the distinction between cortical and peripheral blindness, however they derive their occurrence rates for blindness from Robinson et al. (1987), who reported rates for congenital *peripheral* blindness. Other research that covers both peripheral and cortical blindness suggests that the three leading causes of pediatric blindness in the United States are cortical visual impairment (19%), retinopathy of prematurity (13%), and optic nerve hypoplasia (7%) (Steinkuller et al., 1999). If the leading cause of childhood blindness is cortical visual impairment, one expects that the probability rate of comorbid congenital or early developed cortical blindness with schizophrenia is higher than the probability rate of comorbid congenital or early developed peripheral blindness (say due to retinitis pigmentosa) with schizophrenia. More recent research confirmed these results and ranked cortical visual impairment as the first cause (18%) of childhood blindness in the United States (Kong et al., 2012). If all causes of CB excluding that of cortical origin have a prevalence of 82% in total and if this roughly translates to the probability of comorbid congenital peripheral blindness and schizophrenia being 0.02% according to Silverstein et al. (2013b), the remaining 18% approximately corresponds to 0.004% or 4 out of every 100,000. To put these rates in perspective, Hutchinson-Gilford progeria syndrome is an extremely rare condition (1 out of every 8,000,000) and yet more than 100 relevant case-reports are found in the literature (Pollex and Hegele, 2004).

^2^http://www.who.int/mental_health/management/schizophrenia/en/

^3^The patient received two diagnoses: one was catatonic schizophrenia and the other prolonged partial complex status epilepticus. The clinical features of interictal psychosis of epilepsy are often indistinguishable from schizophrenia (Elliott et al., 2009).

^4^Gender information is not explicit, but inferable.

^5^A reviewer draws our attention to the fact that the research criteria for psychosis have changed substantially over time. Of course, numerous changes can be observed when comparing DSM-I (American Psychiatric Association, 1952) and DSM-V. However, it seems that reference to key features such as hallucinations, catatonia, delusional thinking are found across versions, even if emphasis shifts to different diagnostic markers in the course of time. For aspects of the history of DSM and its influences by various traditions, see Andreasen (2007).

^6^http://www.nidcd.nih.gov/staticresources/health/hearing/FactSheetUsherSyndrome.pdf
